# The Intracellular Virus-Containing Compartments in Primary Human Macrophages Are Largely Inaccessible to Antibodies and Small Molecules

**DOI:** 10.1371/journal.pone.0035297

**Published:** 2012-05-02

**Authors:** Hin Chu, Jaang-Jiun Wang, Mingli Qi, Jeong-Joong Yoon, Xiaoyun Wen, Xuemin Chen, Lingmei Ding, Paul Spearman

**Affiliations:** Department of Pediatrics, Emory University and Children’s Healthcare of Atlanta, Atlanta, Georgia, United States of America; University of Pittsburgh, United States of America

## Abstract

HIV-1 assembly and release occurs at the plasma membrane of human T lymphocytes and model epithelial cell lines, whereas in macrophages intracellular sites of virus assembly or accumulation predominate. The origin of the intracellular virus-containing compartment (VCC) has been controversial. This compartment is enriched in markers of the multivesicular body, and has been described as a modified endosomal compartment. Several studies of this compartment have revealed the presence of small channels connecting to the plasma membrane, suggesting that instead of an endosomal origin the compartment is a modified plasma membrane compartment. If the compartment is accessible to the external environment, this would have important implications for antiviral immune responses and antiviral therapy. We performed a series of experiments designed to determine if the VCC in macrophages was open to the external environment and accessible to antibodies and small molecules. The majority of VCCs were found to be inaccessible to exogenously-applied antibodies to tetraspanins in the absence of membrane permeabilization, while tetraspanin staining was readily observed following membrane permeabilization. Cationized ferritin was utilized to stain the plasma membrane, and revealed that the majority of virus-containing compartments were inaccessible to ferritin. Low molecular weight dextrans could access only a very small percentage of VCCs, and these tended to be more peripheral compartments. We conclude that the VCCs in monocyte-derived human macrophages are heterogeneous, but the majority of VCCs are closed to the external environment.

## Introduction

Human immunodeficiency virus type 1 (HIV-1) assembly occurs predominantly at the plasma membrane of infected T lymphocytes and model epithelial cell lines [1], [2], [3], [4], [5]. In contrast, infected macrophages examined by electron microscopy and immunofluoresent microscopic techniques reveal an intense intracellular accumulation of virions in a compartment marked by characteristic components of the multivesicular body (MVB), including CD81, CD9, MHC Class II, and CD63 [6], [7], [8], [9]. The presence of apparent assembly in intracellular sites with characteristics of the MVB in macrophages led to models for HIV-1 assembly in which the endocytic network plays an important role. Some models for HIV-1 assembly in macrophages propose that intracellular assembly predominates, with release from the intracellular compartment across the virologic synapse upon contact with T cells [10], [11], [12]. This mode of transmission of HIV to T lymphocytes may be essentially the same as that proposed for the dendritic cell-T cell infectious synapse [13], [14], [15]. Defining the precise site of assembly in the macrophage and the factors determining the apparent intracellular assembly site thus has relevance to a number of areas of HIV biology.

**Figure 1:**
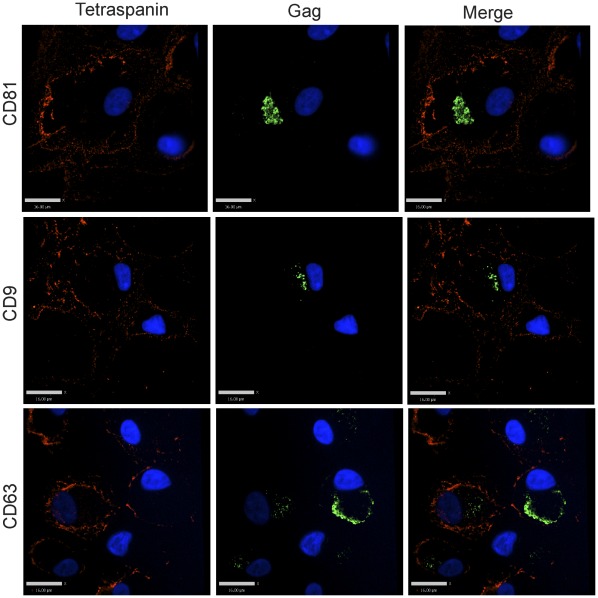
Tetraspanin-enriched HIV-1 positive compartments in infected MDMs are not accessible to the external environment. Human MDMs were infected with VSV-G-pseudotyped HIV-1. 8 days post infection, cells were fixed and immunolabeled for tetraspanins (red, anti-CD81, anti-CD9, or anti-CD63) without cell permeabilization, followed by permeabilization and immunolabeling for HIV-1 Gag (green, anti-MA). Image acquisition was performed with an Improvision/Perkin Elmer spinning disc confocal fluorescence microscope. Bars represent 16 µm.

Small channels linking the VCC in macrophages to the plasma membrane were first identified by Welsch and colleagues using a membrane-impermeant dye ruthenium red [16]. The intracellular VCCs were shown to be accessible from the cell surface by Deneka and colleagues using HRP at 4°C or when fixed and stained with ruthenium red [17]. Images of relatively large conduits extending from intracellular VCCs were demonstrated by Bennett and coworkers [18]. These investigators found that channels of 150–200 nm in diameter led to the cell surface from the VCC. The channels were often found to contain viruses, suggesting that viruses may be directionally released through these channels without invoking exocytosis of the compartment itself. Other investigators report channels that form a complex intracellular network bearing plasma membrane connections that are too small to allow transit of virions [19].The VCC has recently been referred to as the intracellular plasma membrane-connected compartment (IPMC) in recognition of its unique connection to the outside of the cell [20]. The IPMC is a compartment present in cultured macrophages in the absence of HIV-infection, and is characterized by enrichment of the β2 integrin CD18 as well as CD11a, CD11b, talin, vinculin, and paxillin [20].

While it is clear that a proportion of intracellular VCCs contain channels to the external environment, it is not clear that this is a characteristic of all VCCs. In the initial report using ruthenium red, unstained intracellular compartments with virus were equally prominent, and the relative number of stained vs. unstained compartments varied by donor [16]. Another report found that only 20% of apparent endosomal VCCs were stained with ruthenium red [21]. The presence of a VCC that is not accessible would have important potential implications for the ability of macrophages to serve as an HIV reservoir, as viruses in this compartment would be protected from neutralizing antibodies. Furthermore, the presence of an open channel to the exterior of the cell could potentially serve as a route of exit of infectious viral particles or a route of entry of neutralizing antibodies. In this study, we quantified the accessibility of the intracellular VCC in human monocyte-derived macrophages to antibodies, to cell surface staining with cationized ferritin, and to entry of low molecular weight dextrans. We report that the majority of intracellular VCCs are inaccessible to antibodies and dextran and to a general cell surface stain.

**Figure 2:**
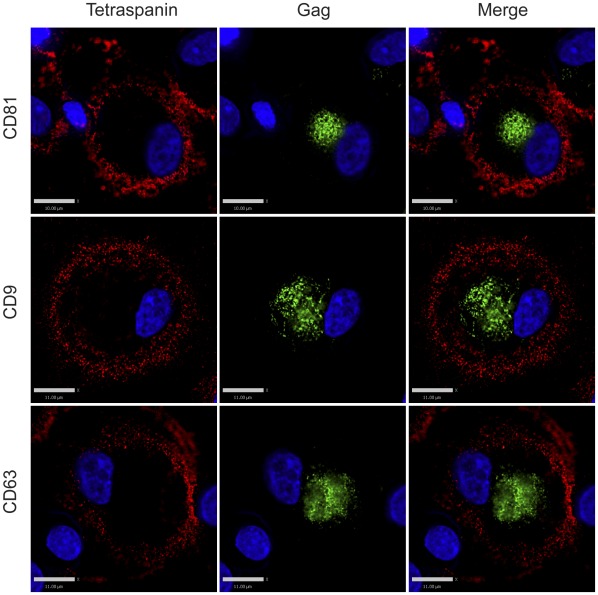
Tetraspanin-enriched HIV-1 positive compartments in infected MDMs are not accessible to the external environment even prior to the fixation procedure. Human MDMs were infected with VSV-G-pseudotyped HIV-1. 8 days post infection, cells were labeled with primary antibodies against tetraspanins (red, anti-CD81, anti-CD9, or anti-CD63) at 4°C for 1.5 hour. Labeled MDMs were then fixed, permeabilized and immunolabeled for secondary antibody against the tetraspanins and HIV-1 Gag (green, anti-MA). Image acquisition was performed with an Applied Precision Deltavision deconvolution microscope. Bars represent 10 µm for the top panels and 11 µm for the middle and bottom panels.

**Figure 3:**
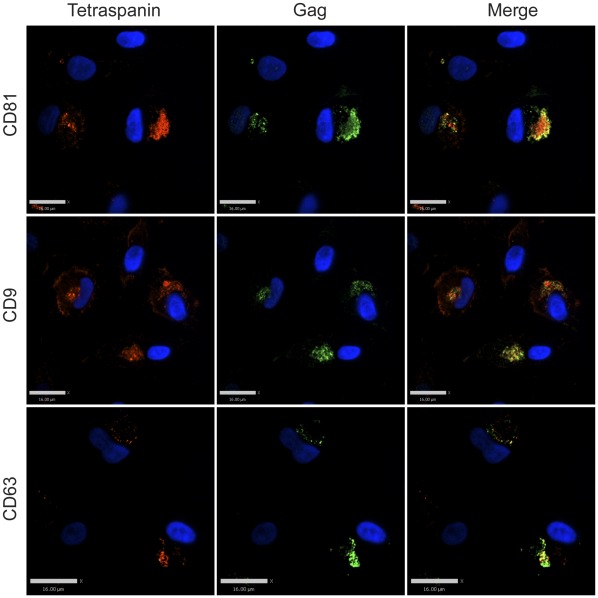
Tetraspanin-enriched HIV-1 positive compartments in infected MDMs are not accessible to the external environment until permeabilized. Human MDMs were infected with VSV-G-pseudotyped HIV-1. 8 days post infection, cells were fixed and immunolabeled for HIV-1 Gag (green, anti-MA) and tetraspanins (red, anti-CD81, anti-CD9, or anti-CD63) after permeabilization, followed by imaging acquisition with an Improvision/Perkin Elmer spinning disc confocal fluorescence microscope. Bars represent 16 µm.

## Results

### Accessibility of Tetraspanin-rich HIV-1 Containing Compartments in MDMs to Antibodies

It has been previously demonstrated that the virus-containing compartments (VCCs) in infected macrophages are enriched in tetraspanins including CD81, CD82, CD9, CD63, and in MHC class II molecules [6], [7], [8], [9], [17]. We first endeavored to establish in our hands whether the VCCs were contiguous with the plasma membrane and open to the external environment as has been demonstrated by others [17], [18]. To do this, we tested the ability of antibodies against CD81, CD9, and CD63 to stain the compartments before and after cell permeabilization. Antibodies were directed against the extracellular loops of the tetraspanins, which are oriented toward the lumen of the VCC and should be accessible to an antibody reaching this compartment. HIV-infected monocyte-derived macrophages (MDMs) were fixed and immunolabeled with antibodies against either CD81, CD9, or CD63 in the absence of cell permeabilization, followed by cell permeabilization and labeling with an anti-Gag antibody. We observed significant labeling of the tetraspanins at the plasma membrane under these conditions, but not within intracellular compartments of the cells in the absence of permeabilization (Fig. 1). The majority of Gag protein staining was observed in intracellular compartments, and we did not observe colocalization between tetraspanins and Gag staining in unpermeabilized cells. To examine the possibility that the fixation process might have affected the accessibility of the antibodies to the VCCs by altering or cross-linking the channels, we labelled infected MDMs at 4°C prior to any fixation, then fixed, permeabilized, and stained for Gag protein as before. Tetraspanins were again detected on the plasma membrane of infected MDMs, and Gag was readily detected in the VCCs, while no anti-tetraspanin antibody was observed within this intracellular compartment (Fig. 2). In contrast, HIV-infected MDMs that were first permeabilized and then immunolabeled with antibodies against tetraspanins displayed prominent tetraspanin labeling both at the plasma membrane and within the intracellular compartments. Permeabilization revealed a prominent population of tetraspanin that colocalized with Gag in large intracellular VCCs (Fig. 3). These experiments indicated to us that antibodies could not access the VCCs in intact MDMs.

**Figure 4:**
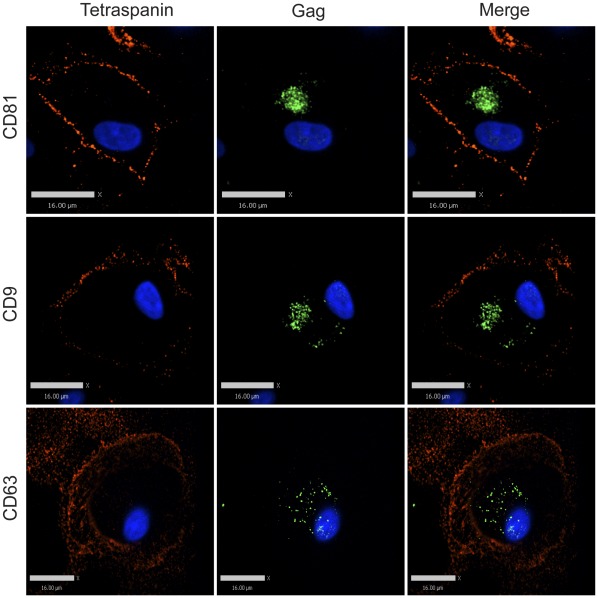
Tetraspanin-enriched HIV-1 positive endosomal compartments in infected MDMs are not accessible to the external environment. Human MDMs were infected with VSV-G-pseudotyped 29/31 KE mutant HIV-1. 8 days post infection, cells were fixed and immunolabeled for tetraspanins (red, anti-CD81, anti-CD9, or anti-CD63) without cell permeabilization, followed by permeabilization and immunolabeling for HIV-1 Gag (green, anti-MA). Image acquisition was performed with an Improvision/Perkin Elmer spinning disc confocal fluorescence microscope. Bars represent 16 µm.

**Figure 5:**
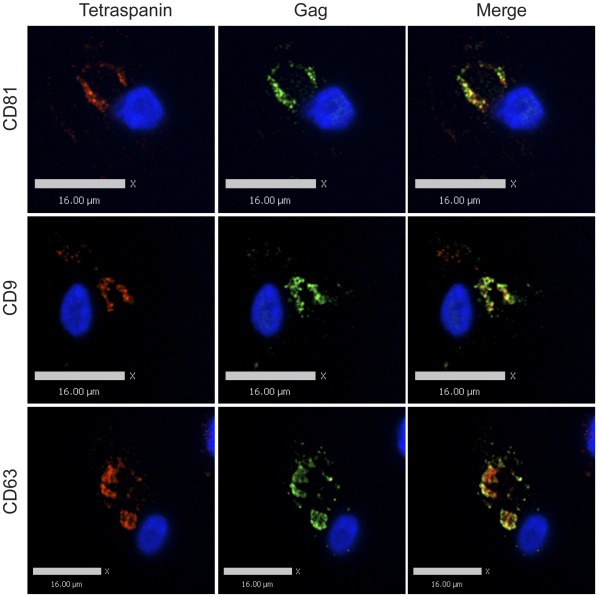
Endosomal compartment markers colocalize with Gag in macrophages infected with 29/31 KE endosomal-targeting mutant virus after permeabilization. Human MDMs were infected with VSV-G-pseudotyped 29/31 KE mutant HIV-1. 8 days post infection, cells were fixed and immunolabeled for HIV-1 Gag (green, anti-MA) and tetraspanins (red, anti-CD81, anti-CD9, or anti-CD63) after cell permeabilization, followed by imaging acquisition with an Improvision/Perkin Elmer spinning disc confocal fluorescence microscope. Bars represent 16 µm.

We next utilized a previously described MA mutant, 29/31KE, that assembles within the intracellular MVB compartment as a tool to determine if the compartment in macrophages in which we observed Gag and tetraspanin colocalization was indeed endosomal in nature [22], [23]. MDMs were infected with VSV-G-pseudotyped HIV 29/31KE virus for 8 days, followed by immunostaining for CD81, CD9, CD63, and Gag. As we had observed with wildtype virus-infected cells, for cells labeled with antibodies against the tetraspanins before cell permeabilization, minimal intracellular tetraspanin label and little colocalization between tetraspanins and Gag was observed (Fig. 4). In contrast, when cells were first permeabilized, a significant amount of tetraspanin staining was observed intracellularly, and cells displayed a strong colocalization between tetraspanins and intracellular Gag in general (Fig. 5). Thus the MVB-targeted mutant virus identified an intracellular compartment that appeared identical to that marked by wildtype virus, and this compartment was not accessible to antibodies applied externally.

**Figure 6:**
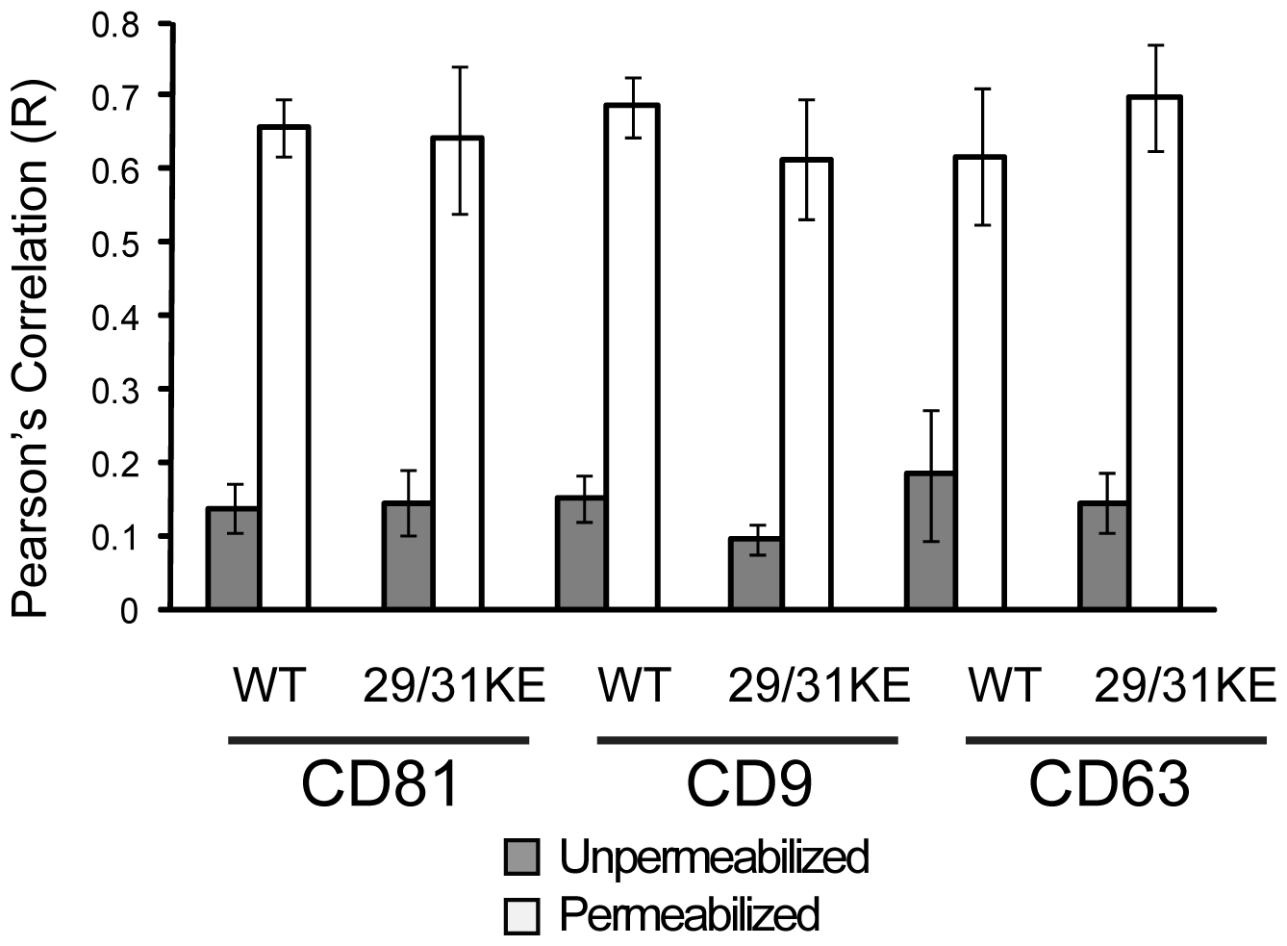
Quantitation of colocalization of Gag in macrophages infected with WT or 29/31 KE endosomal-targeting mutant virus before and after permeabilization. Volocity software package (Perkin Elmer) was used to quantify colocalized pixels before (grey) and after (white) permeabilization of cells. Error bars represent standard deviation, from a total of 30 cells examined for each experiment.

To account for cell- to cell-variation across different donors and provide quantitation of colocalization, we compared the degree of colocalization between the tetraspanins and Gag in MDMs quantitatively using cells from five individual donors. We employed images of 30 cells stained for Gag and each individual tetraspanin to derive the colocalization data shown in Fig. 6. For cells in which tetraspanin staining was performed before cell permeabilization, the average R-value (using Pearson’s correlation) for tetraspanin and Gag colocalization for wildtype virus was 0.14 ±0.03, 0.15 ±0.03, and 0.18±0.09 for CD81, CD9, and CD63, respectively. For cells in which tetraspanin staining was performed at 4°C before fixation and permeabilization, we detected an averaged R-value of 0.14 ±0.02, 0.14 ±0.03, and 0.13 ±0.03 for CD81, CD9, and CD63, respectively (data not shown). For cells in which tetraspanin staining was performed after cell permeabilization, we detected an averaged R-value of 0.66 ±0.04, 0.69 ±0.04, and 0.62 ±0.09 for CD81, CD9, and CD63, respectively (quantitation presented in Fig. 6). These results confirmed our visual impression that antibodies to the tetraspanins were generally not able to reach the intracellular VCC in intact non-permeabilized macrophages. Results were quite similar for MDMs infected with virus bearing the 29/31KE mutation (Fig. 6). When tetraspanin staining was performed before cell permeabilization, the average R-value for tetraspanins and Gag colocalization was 0.14 ±0.05, 0.10 ±0.02, and 0.15±0.04 for CD81, CD9, and CD63, respectively. For cells in which tetraspanin staining was performed after cell permeabilization, we detected an averaged R-value of 0.64 ±0.10, 0.61 ±0.08, and 0.70±0.07 for CD81, CD9, and CD63, respectively (Fig. 6). These results indicate that both wildtype and 29/31KE virus are concentrated in an MVB-like compartment within human macrophages that is not accessible to antibodies.

### The Predominant Population of the Virus-containing Compartments in Infected MDMs is Inaccessible to the Cationic Ferritin Cell Surface Label

**Figure 7:**
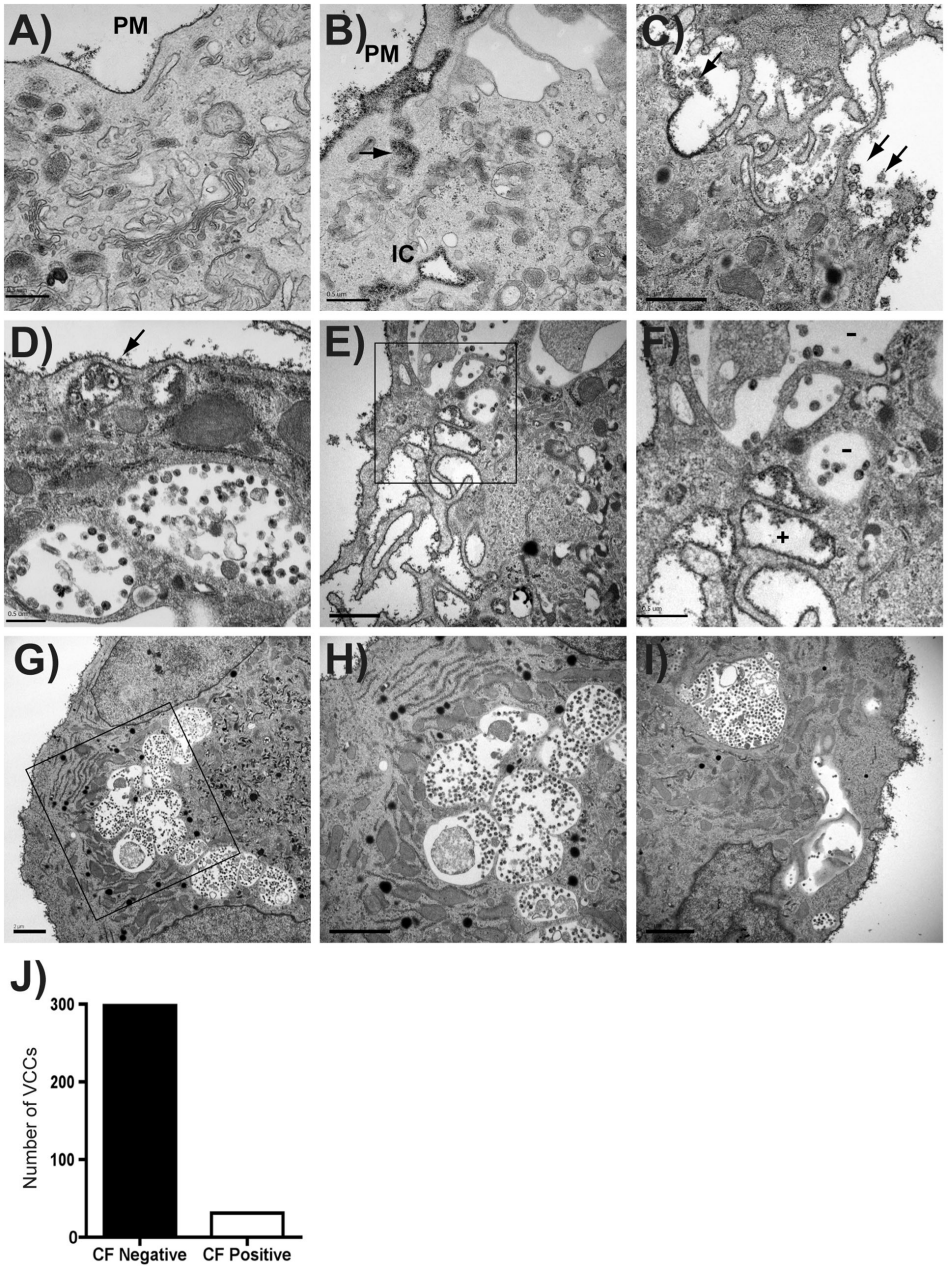
The majority of virus-containing compartments in infected MDMs are inaccessible to a cell surface label. (A-B) Uninfected Human MDMs cultured on ACLAR embedding film were fixed and stained with cationized ferritin (CF), followed by standard electron microscope processing procedures. Images were then obtained under a Hitachi H-7500 transmission electron microscope. Cationized ferritin labeled plasma membrane is seen along the plasma membrane (PM). (A) and (B) represent uninfected macrophages, bars  =  0.5 µm. IC  =  apparent intracellular space stained with CF. (C) HIV particles were seen at PM and stained with CF on periphery of cells (arrows). Bar  =  1 µm. (D) CF is seen staining HIV particles underlying a PM fold (arrow), while deeper VCCs lack CF staining. Bar  =  0.5 µm. (E-F) CF staining of membrane protrusions contrasts with lack of CF in intracellular VCCs (F is higher magnification view of boxed region in E). Bar  =  1.0 µm (E), 0.5 µm (F). (G-I) Additional views of PM staining with CF and exclusion of CF from VCC. (H represents higher magnification view of boxed region from G, bars  =  2 µm). (J) VCCs were counted as CF+ or CF- from 329 apparent intracellular VCCs in more than 50 cells. The number of CF-negative compartments vs. CF-positive compartments is indicated.

To evaluate the nature of the assembly compartments using another approach, we employed electron microscopy (EM) and cationized ferritin staining, which provided a direct visualization of the cell surface and potentially the structure of the assembly compartments independent of immunolabeling. Cationized ferritin (CF) is a positively charged membrane-impermeable dye that has been used to study the ultrastructure of the cell membrane [24], [25]. We first established the ferritin staining method in uninfected MDMs. In these cells, we observed strong labeling of cationized ferritin on the plasma membrane (Fig. 7A, 7B). In addition, we detected apparent intracellular compartments that were positively labeled with ferritin, suggesting that these compartments were in fact accessible from the plasma membrane, similar to findings of other groups [16], [17] (Fig. 7B, IC). CF-stained folds or channels were apparent in connection with these stained compartments and with the plasma membrane (Fig. 7B). Thus, CF stained the plasma membrane of MDMs and reproduced the finding of folded PM or small channels that had been described by others.

We next applied plasma membrane staining with CF to HIV-infected MDMs for a quantitative analysis of staining of the VCC. To do this, mature MDMs cultured on ACLAR embedding film were infected with HIV-1, maintained in macrophage growth media for eight days, fixed with paraformaldehyde/glutaraldehyde, and stained with cationized ferritin. Infected macrophages demonstrated virus particles at the plasma membrane that were stained with CF (Fig. 7C). As with uninfected MDMs, we found both ferritin-positive and ferritin-negative compartments in infected MDMs (Fig. 7D). We observed that cationized ferritin labeled the folded plasma membrane and some apparent intracellular compartments, in particular those near the periphery of cells (Fig. 7D, arrow), while larger VCCs were often unlabeled with CF (Fig. 7D, lower compartments). Fig 7E and 7F illustrate CF labeling of the PM protrusions of the macrophage along with unlabeled VCCs (minus signs indicate CF-negative compartments in 7F). Fig. 7G-I provide additional representative sections demonstrating cell surface CF staining and CF-negative intracellular VCCs. We then quantified the number of surface label-positive and negative intracellular VCCs in infected MDMs from 5 different donors. Altogether, we identified 329 intracellular viral compartments in 51 cells, and of these 298 or 90.6% were ferritin-negative and only 31 or 9.4% were ferritin positive (Fig. 7J). We conclude from these results and those from antibody labeling experiments described above that the majority of the deep, intracellular VCCs are inaccessible to the extracellular environment, while a minority of smaller, more peripheral virus-containing compartments with connection to the plasma membrane are also present.

**Figure 8:**
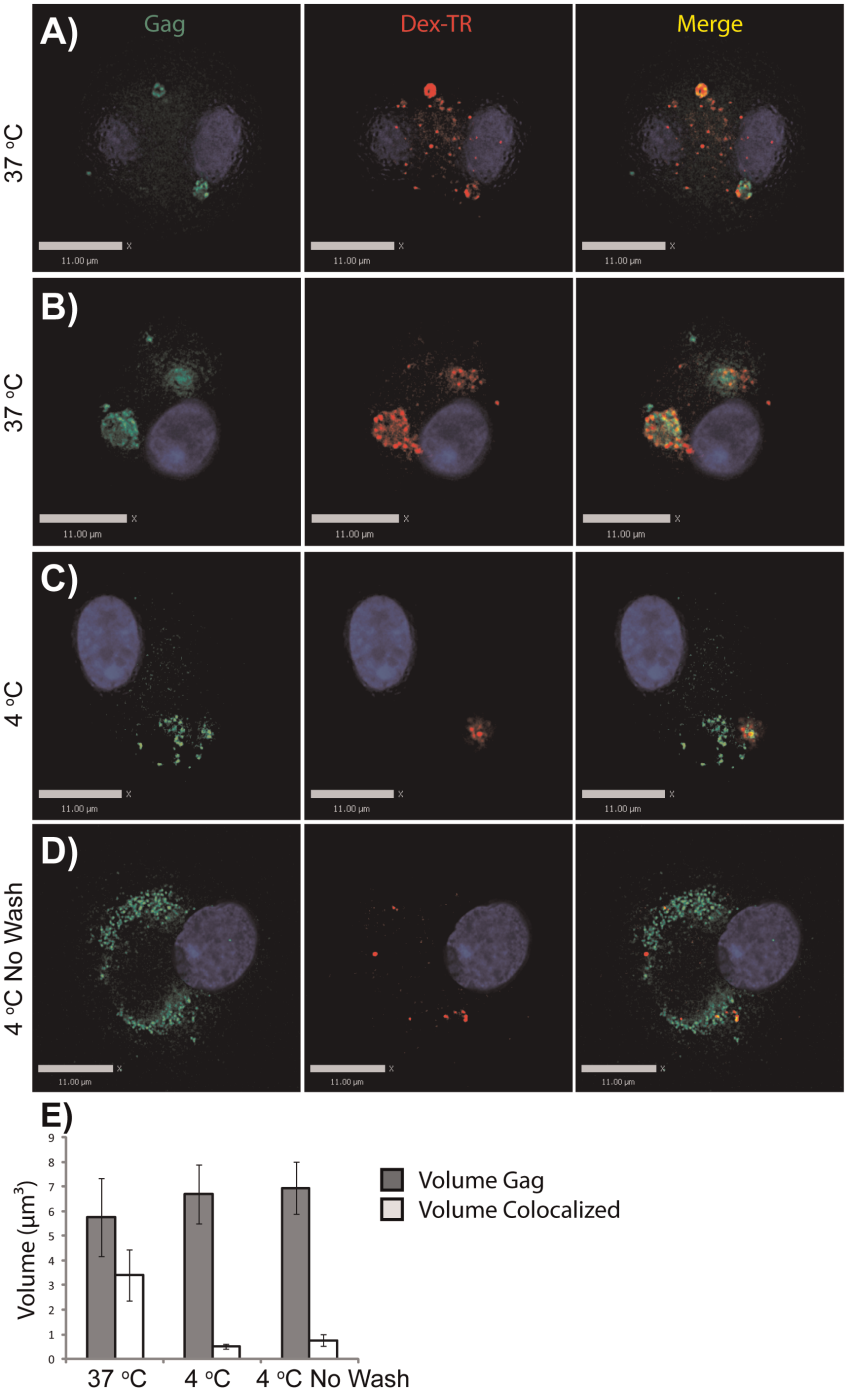
Low molecular weight dextran is largely excluded from VCCs in macrophages. HIV-infected MDMs were incubated with Texas red dextran, 3000 MW, at 37°C or 4°C for 30 minutes. Cells were then fixed and stained for Gag (green). (A-B) Representative images of cells incubated at 37°C. (C) Representative image of cells incubated at 4°C. (D) Representative image of cells incubated at 4°C with no wash prior to fixation. Bars  =  11 µm. (E) Quantitation of colocalized voxels from 3D image stacks derived from ten cells at each temperature, presented as µm^3^. Error bars indicate standard deviation.

### Low Molecular Weight Dextrans are Largely Excluded from VCCs in HIV-1-Infected MDMs

We considered that antibodies are relatively large structures that may not be able to pass through very narrow plasma membrane channels, especially if the channels are formed by folds in which membranes are very closely apposed. Ferritin is also a large cation (450 kD) and might not pass through convoluted, narrow channels. We therefore next examined the ability of fluorescent low-molecular weight dextran to reach the VCC in unfixed MDMs. HIV-infected MDMs were incubated at 37°C or 4°C with Texas Red-Dextran (Dex-TR, 3000 MW) for 30 minutes prior to fixation, permeabilization, and staining as before. MDMs incubated at 37°C for 30 minutes demonstrated strong colocalization of dextran and Gag in intracellular compartments (Fig. 8A and 8B). On the other hand, colocalization of dextran and Gag in VCCs at 4°C was much more limited regardless of the washes prior to fixation (Fig. 8C). In order to ensure that we were not washing out the dextran in this experiment, we simply aspirated and replaced the media without any additional wash steps. The colocalization of dextran and Gag remained very limited even in the absence of washes (Fig. 8D). Intriguingly, we observed some dextran-labeled compartments that were in close proximity to the VCCs but did not colocalize with Gag (Fig. 8C and 8D). Dextran-positive compartments at 4°C were in general small and peripheral (Fig. 8C and 8D), similar to the CF-positive VCCs identified in our transmission EM studies. We acquired z-stacks of images from ten representative cells at each temperature, and determined colocalization by volume of intracellular Gag and intracellular dextran at these temperatures. Fig. 8E shows the comparison of intracellular Gag volume (grey bars) to the volume where dextran and Gag colocalized, presented as averaged intracellular Gag voxels/cell (gray bars) and averaged colocalized (Gag + dextran) voxels/cell (white bars). We note that at 4°C, colocalization of intracellular Gag and dextran was quite low (0.51 µm^3^ to 0.77 µm^3^ colocalized voxels on average per cell, versus 3.40 µm^3^ colocalized voxels at 37°C, Fig. 8D). Thus, despite 30 minutes of contact with living cells at 4°C, little dextran reached the VCC. These results, together with the antibody and CF results above indicate that the majority of VCCs are not open and accessible to the external environment.

## Discussion

Intracellular membrane-bound compartments harboring large collections of virus particles are frequently observed in HIV-1 infected macrophages. These compartments are variably referred to as virus-containing compartments (VCCs) or virus assembly compartments, because in addition to mature virions, immature virus particles as well as active budding viral structures are sometimes observed [18], [26]. Despite an intense investigation into the characteristics of these intracellular compartments in macrophages, the nature and origin of the compartments remains incompletely understood. It is clear that some proportion of the intracellular virion-containing compartments contain thin connections or tubules linked to the exterior of the cell [16], [17], [18]. In the current study, our data argue that although surface accessible compartments are present in infected MDMs, the majority of the VCCs are not open to the external environment, and those that are connected appear to be located peripherally in macrophages. It is possible as noted by others that the antibody molecules might be too large to go through the folded membrane channels [17]. However, CF staining in our experiments similarly failed to demonstrate a connection to the plasma membrane for the majority of virus-containing compartments, and even low molecular weight dextran failed to reach the deep VCCs. Our results suggest that the majority of large intracellular collections of virions in MDMs reside within a modified endosomal compartment that is discontinuous with the surface of the cell. These results are most consistent with recent reports from the Benaroch laboratory [21], [27] defining a non-acidic endosomal compartment with only a minority of the compartments bearing connections to the plasma membrane.

The reason for differences between our findings and those of Deneka and colleagues [17] are not entirely clear. CF is a well-established plasma membrane stain that has been utilized to stain the surface of macrophages and other cell types [24], [28], [29]. Ferritin is a 450 Kd protein and could be excluded from very small channels, and this could explain some lack of access to intracellular compartments in which a channel connects to the PM. It seems unlikely that ferritin would not pass easily through the 150–200 nm open channels described by Bennett and colleagues [18]. We expected that low molecular weight dextrans (3 Kd) would reach the VCCs if open connections were present to the external media. Although dextrans were efficiently taken into the VCC within 30 minutes at 37°C, they were not found in the majority of VCCs at 4°C. We conclude that the majority of the VCCs are not accessible to the surrounding media, so that if there are channels to these VCCs they are effectively closed. We think this finding is not inconsistent with those reports utilizing the cationic tracer ruthenium red to define channels to the plasma membrane, where ruthenium red-inaccessible VCCs are also observed deep within the cell [16], [19].

If channels exist connecting all VCCs to the plasma membrane, as suggested by the model in which the VCC is synonymous with the IPMC [20], then an active process not occurring at 4°C appears to be required to open the channels. The surface connections may be highly dynamic in living cells, with active ruffling and folding of plasma membrane and potentially opening of narrow channels. We could not in the current study distinguish this possibility from active fluid-phase endocytosis or pinocytosis and delivery into a closed VCC lacking all surface connections.

Macrophages are thought to represent an important reservoir of HIV in infected individuals [27], [30], [31]. Intracellular VCCs could protect virus from recognition by antibodies and prevent neutralization or attachment of binding, non-neutralizing antibodies. The finding here that the majority of VCCs are not accessed by antibodies in culture supports this notion of a protected compartment. We note that a recent publication from the Schindler laboratory demonstrated findings entirely compatible with those in our report, including lack of access of antibodies to this closed compartment [32]. In addition to the lack of accessibility of the VCC to anti-CD81 antibodies, they demonstrate that neutralizing antibodies to HIV-1 gp120 failed to reach the VCC in the absence of permeabilization. Thus, the data with cationized ferritin staining, accessibility to dextran, and accessibility to antibodies directed against tetraspanins and against gp120 all indicate that the majority of VCCs in macrophages are effectively closed compartments.

We found viral particles on the plasma membrane of macrophages, which was not the major goal of the study but is contrary to the idea that particle release from the surface of macrophages is rarely observed. These particles were readily stained with the CF surface marker as shown in Fig. 6C. Our findings in this regard are not unique [16] but serve to reinforce the fact that budding occurring on the plasma membrane surface of macrophages is not a rare event and can be captured in EM images of infected human macrophages.

In summary, we detected connections between the external environment and the VCC in only a minority of compartments using a variety of labeling techniques. Antibodies, cationized ferritin, and low molecular weight dextran were excluded from the majority of VCCs. Our results suggest that the majority of VCCs in macrophages are not open to the external environment but rather form a closed intracellular space.

## Materials and Methods

### Ethics Statement

Peripheral blood was obtained from healthy adult volunteer donors according to a protocol approved by the Emory University Institutional Review Board (IRB). The Emory IRB approval applies specifically to this study utilizing de-identified blood samples, as well as other studies using de-identified blood samples that were listed under the same protocol. Written informed consent was obtained from donors, and samples were de-identified prior to handling by laboratory personnel.

### Preparation of Monocyte-derived Macrophages (MDMs)

Peripheral blood mononuclear cells (PBMCs) were obtained from the buffy coat after Ficoll centrifugation and were allowed to adhere to plastic surface coated with poly-D-lysine (Sigma Aldrich, Saint Louis, MO, USA). Nonadherent PBMCs were washed away after 1 hour. Adherent monocytes were maintained in RPMI-1640 supplemented with 10% FBS, 100 ug/ml streptomycin, 100 U/ml penicillin, 2 mM glutamine, 1% sodium pyruvate, 1% non-essential amino acids, and 1U/ml GM-CSF (Cell Sciences, Canton, MA, USA). Monocytes were maintained in the supplemented media for 8 days for differentiation into macrophages, during which the media was replaced every 2 days. The purity of the macrophage population was assessed at day 10 by CD14 staining and was greater than 93%.

### Antibodies and Immunostaining Reagents

Mouse monoclonal antibodies against CD81, CD9, and CD63were obtained from BD Biosciences (San Jose, CA, USA). HIV Gag detection was performed with either rabbit anti-p17 polyclonal [33], mouse anti-p24 monoclonal CA-183 (provided by Bruce Chesebro and Kathy Wehrly through the NIH AIDS Research and Reference Reagent Program), or mouse anti-p24-FITC (KC57-FITC) obtained from Beckman Coulter (Fullerton, CA, USA). Alexa Fluor goat anti-mouse and Alexa Fluor goat anti-rabbit secondary antibodies, as well as the DAPI nucleic acid stain were obtained from Molecular Probes (Eugene, OR, USA).

### Virus Stocks and Infections

Vesicular stomatitis virus G glycoprotein (VSV-G)-pseudotyped HIV-1 NL4-3 virus stocks were generated by cotransfecting 293T cells with pNL4-3 and the VSV-G expression plasmid pHCMV-G [34]. VSV-G-pseudotyped HIV-NL4-3/29/31KE virus stocks were generated by cotransfecting 293T cells with pNL4-3/29/31KE and pHCMV-G. The pNL4-3/29/31KE construct was kindly provided by Dr. Eric Freed (National Cancer Institute, Frederick, MD) and has been described previously [23]. Viruses were harvested from transfected 293T supernatants 48 hours post-transfection, filtered through 0.45 µm filters, and assayed with TZM-βl indicator cells for infectivity assessment. For infections, MDMs were incubated with VSV-G-pseudotyped virus stocks or with non-pseudotyped BaL biological stock at 0.5 50% tissue culture infectious dose (TCID_50_) per cell for 4 hours. Cells were then washed with PBS and incubated for 0 to 12 days before harvesting for analysis.

### Image Acquisition and Analysis

Two imaging stations equipped for live cell imaging were employed in this study. The first imaging station was a Nikon TE2000-U spinning disc confocal fluorescence microscope with automated stage and Hamamatsu EM-CCD camera developed by Improvision under the control of the Volocity software. The second imaging station was a Deltavision imaging system developed by Applied Precision. The system was equipped with an Olympus IX70 microscope and a CoolSnap HQ2 digital camera under the control of the softWoRx software. Imaging processing and deconvolution was performed using softWoRx 3.7.0. Colocalization and partial colocalization measurements were quantified with the colocalization module of Volocity 5.2.1. The volume and the intensity of Gag among the colocalized pixels were calculated using the measurements module of Volocity 5.5.1. Volocity 5.2.1, Volocity 5.5.1, Adobe Photoshop CS4 and Adobe Illustrator CS4 were used to analysis and adjust the images.

### Immunofluorescence Microscopy

MDMs were washed with PBS and fixed in 4% paraformaldehyde for 12 minutes at RT. After fixation, cells were extensively washed including an overnight wash at 4°C. Cells were then permeabilized for 10 minutes with 0.2% Triton X-100 and blocked in Dako blocking buffer for 30 minutes. Primary and secondary antibodies were diluted in Dako antibody diluent to appropriate concentrations. In general, cells were stained in primary antibodies for 1.5 hours and in secondary antibodies for 45 minutes. DAPI was used to stain the nuclei of the cells. The coverslips were mounted in Gelvatol overnight and examined directly the next day. To label the infected MDMs at 4°C prior to fixation, the cells were washed with cold PBS and cooled on ice for 30 minutes. Primary antibodies against tetraspanins were then added to the MDMs and were incubated with MDMs at 4°C for 1.5 hour. Labeled MDMs were then washed with PBS and fixed in 4% paraformaldehyde for 12 minutes. After fixation, the cells were permeabilized, blocked, and stained for secondary antibody for the tetraspanins and Gag as described above.

### Electron Microscopy

For cationized ferritin labeling, MDMs cultured on ACLAR embedding film (Ted Pella, Redding, CA, USA) were washed with PBS and fixed in 2.5% paraformaldehyde and 2.5% glutaraldehyde in 0.1 M sodium cacodylate buffer (EMS, Hatfield, PA, USA), pH 7.4 for 1 hour at RT. After washing in 0.1 M sodium cacodylate buffer with 6% sucrose, the cells were then post-fixed with 1% osmium (OsO_4_) for 1 hour on ice and labeled with 1 mg/ml cationized ferritin (Sigma Aldrich, St. Louis, MO, USA) for 1.5 hour at RT. CF-labeled samples were en-block stained with aqueous uranyl acetate, gradient dehydrated, filled with epon, embedded in Chang’s flat-embedding cambers and processed for thin sectioning. Images were obtained under a Hitachi H-7500 transmission electron microscope at 75 KV.

### Low Molecular Weight Dextran Accessibility Experiments

MDMs were differentiated for 7 days and were subsequently infected with VSV-G-pseudotyped NL4-3 for 8 days. Infected MDMs were then treated with 0.5 mg/ml lysine fixable Texas red dextran (dex-TR, 3000 MW, Molecular Probes, Eugene, OR, USA) for 30 minutes at 37°C or at 4°C. For studies at 37°C, MDMs were washed with PBS, followed by adding MDM growth media containing 0.5 mg/ml dex-TR. The cells were returned to the incubator and incubated for 30 minutes. Dex-TR label MDMs were then extensively washed with PBS and fixed with 4% paraformaldehyde for 15 minutes. For studies at 4°C, MDMs were first cooled on ice for 30 minutes. The cells were then washed with ice-cold PBS, followed by adding ice-cold MDM growth media containing 0.5 mg/ml dex-TR. The cells were then incubated at 4°C for 30 minutes. Dex-TR label MDMs were then washed with ice-cold PBS and fixed with cold 4% paraformaldehyde for 15 minutes. In parallel, a group of labeled MDMs were fixed directly in cold 4% paraformaldehyde after aspirating the dex-TR labeling media in the absence of washes. Fixed MDMs were subsequently immunolabeled for HIV-1 Gag as described earlier.
